# Without Encumbrance

**Published:** 1912-08-24

**Authors:** 


### "WITHOUT ENCUMBRANCE."

Registrars and statisticians have for years been commenting on the steady and serious fall in the National birth-rate, though sociologists like Havelock Ellis have done much to show that a "crude birth-rate" leads to fallacious and ill-considered alarm. The Colonies provide outlets for the energies of many for whom the humdrum existence of a profession or a trade has little attraction. In a word, the middle classes have solid grounds for their boast that they are the backbone of England. It is this class which finds itself most seriously handicapped by the changing conditions of modern society. Unfortunately for the State, it is this class which is restricting its birth-rate most of all. Thus the loss of future citizens is greatest among the very section of society whose offspring are most likely to prove valuable members of the community.

Momentous as this situation is, its consideration holds out no immediate prospect of vote-catching, so neither political party will devote any serious attention to it. Various remedies, such as the taxation of bachelors, have been suggested, but nothing is done. Nay, worse; both private and public employers are allowed to foster the canker by making it impossible for those under their control to have large families, or even, in many cases, to have families at all. All too often is an advertisement published of a post of some kind for a man or a woman or a married couple, with the condition that they must be either single or (if married) "without encumbrance." Now this state of things reflects the gravest discredit on the whole nation, but especially on those who claim to represent it—namely, Parliament, pulpit, and press. Only a few days ago the Kingston-on-Thames Guardians were very justly pilloried for this offence. The positions advertised were those of workhouse master and matron, to be held by a married couple "without encumbrance." The Vice-Chairman, in his defence of the Board's advertisement, admits the crime, but defends it on grounds of expediency. He says that a workhouse is not a "desirable place in which to bring up a young family," and that both master and matron should "be able to give undivided attention to their work."

Anything less satisfactory, less humane, less public-spirited, the imagination fails to conceive. In order that the supervision of workhouse inmates may be properly carried out, those who undertake it for the Guardians must be denied the privileges of paternity and maternity! The answer to this disgraceful attitude is supplied by the National Poor-Law Officers' Association in a circular just issued, which reveals that it is exceptional for any proper provision to be made for married officials, and that in many cases to become a father jeopardises a man's position and ruins his prospects! The recommendations of the Association for the abolition of these scandals seem to be reasonable and practical; it is to be hoped that Boards of Guardians will consider them. As members of the middle classes, their sympathies should be with, not against, the difficulties of Poor-Law employees in respect of family ties. Many Boards of Guardians include clergymen of all denominations; cannot they do something to put an end to the crimes against the State, of which the "without encumbrance" advertisement is the type? Anything more preposterous than the argument above quoted can hardly be imagined.

